# Supplementary material for: Influence of Extracellular Matrix Components on the Differentiation of Periodontal Ligament Stem Cells in Collagen I Hydrogel
Source: Cells. 2023 Sep 22;12(19):2335. doi: 10.3390/cells12192335 (PMC10571948; doi:10.3390/cells12192335)
Supplement: Supplementary file 1 [file cells-12-02335-s001.zip › cells-2537480-supplementary.pdf]

Table S1: Data of morphometric analysis of expression of CD44 in PDLSCs cultured under 3D conditions for 14 days. Anova: One-way analysis of variance with Tukey-Kramer test for all pairwise comparisons.

One-way analysis of variance

Data	% of cells
Factor codes	CD44

Sample size	40

**Levene's test for equality of error variances**	
Levene statistic	1.679
DF 1	7
DF 2	32
Significance level	P = 0.150

ANOVA

Source of variation	Sum of Squares	DF	Mean Square
Between groups (influence factor)	66954.3792	7	9564.9113
Within groups (other fluctuations)	29.8125	32	0.9316
Total	66984.1917	39	

F-ratio	10266.726
Significance level	P < 0.001

Tukey-Kramer test for all pairwise comparisons

Factor	n	Mean	SD	Different (P<0.05) from factor nr
(1) 1	5	77.3089	1.463	(2)(3)(4)(5)(6)(7)(8)
(2) 2	5	89.5875	0.9166	(1)(3)(4)(5)(6)(7)(8)
(3) 3	5	20.4112	1.1358	(1)(2)(4)(5)(6)(7)(8)
(4) 4	5	0.0000	0.0000	(1)(2)(3)(5)(6)(7)
(5) 5	5	94.2816	1.0853	(1)(2)(3)(4)(8)
(6) 6	5	94.2805	1.0365	(1)(2)(3)(4)(8)
(7) 7	5	93.8981	0.6404	(1)(2)(3)(4)(8)
(8) 8	5	0.3226	0.7213	(1)(2)(3)(5)(6)(7)

Residuals

D'Agostino-Pearson test for Normal distribution	accept Normality (P=0.2638)

Table S2: Data of morphometric analysis of expression of STRO-1 in PDLSCs cultured under 3D conditions for 14 days. Anova: One-way analysis of variance with Tukey-Kramer test for all pairwise comparisons.

**One-way analysis of variance**				
Data	% of cells			
Factor codes	STRO-1			
Sample size	40			
**Levene's test for equality of error variances**				
Levene statistic	3.637			
DF 1	7			
DF 2	32			
Significance level	P = 0.005			
**ANOVA**				
Source of variation	Sum of Squares	DF	Mean Square	
Between groups (influence factor)	66369.671	7	9481.3816	
Within groups (other fluctuations)	60.6645	32	1.8958	
Total	66430.3354	39		
F-ratio	5001.35			
Significance level	P < 0.001			
**Tukey-Kramer test for all pairwise comparisons**				
Factor	n	Mean	SD	Different (P<0.05) from factor nr
(1) 1	5	73.1858	2.2388	(2)(3)(4)(5)(6)(7)(8)
(2) 2	5	90.1822	2.3244	(1)(3)(4)(5)(6)(7)(8)
(3) 3	5	22.1966	1.784	(1)(2)(4)(5)(6)(7)(8)
(4) 4	5	0.137	0.3063	(1)(2)(3)(5)(6)(7)
(5) 5	5	94.5986	0.837	(1)(2)(3)(4)(8)
(6) 6	5	94.5331	0.4317	(1)(2)(3)(4)(8)
(7) 7	5	94.6504	0.7664	(1)(2)(3)(4)(8)
(8) 8	5	0.0000	0.0000	(1)(2)(3)(5)(6)(7)
**Residuals**				
D'Agostino-Pearson test for Normal distribution	reject Normality (P=0.0223)			

Table S3: Data of morphometric analysis of expression of OC in PDLSCs cultured under 3D conditions for 14 days. Anova: One-way analysis of variance with Tukey-Kramer test for all pairwise comparisons.

One-way analysis of variance

Data	% of cells
Factor codes	OC
Sample size	40

Levene's test for equality of error variances

Levene statistic	1.023
DF 1	7
DF 2	32
Significance level	P = 0.434

ANOVA

Source of variation	Sum of Squares	DF	Mean Square
Between groups (influence factor)	5989.0828	7	855.5833
Within groups (other fluctuations)	26.3951	32	0.8248
Total	6015.4779	39	

F-ratio	1037.264
Significance level	P < 0.001

Tukey-Kramer test for all pairwise comparisons

Factor	n	Mean	SD	Different (P<0.05) from factor nr
(1) 1	5	60.5988	1.5746	(2)(3)(4)(5)(6)(7)(8)
(2) 2	5	73.8089	0.8986	(1)(3)(4)(5)(6)(7)(8)
(3) 3	5	89.9246	0.7972	(1)(2)(5)(6)(7)(8)
(4) 4	5	90.4936	0.5665	(1)(2)(5)(6)(7)(8)
(5) 5	5	93.0215	0.6424	(1)(2)(3)(4)(6)(7)(8)
(6) 6	5	94.9863	1.173	(1)(2)(3)(4)(5)(7)
(7) 7	5	98.44	0.2267	(1)(2)(3)(4)(5)(6)(8)
(8) 8	5	95.6734	0.7178	(1)(2)(3)(4)(5)(7)

Residuals

D'Agostino-Pearson test for Normal distribution	accept Normality (P=0.2721)

Table S4: Data of morphometric analysis of expression of OPN in PDLSCs cultured under 3D conditions for 14 days. Anova: One-way analysis of variance with Tukey-Kramer test for all pairwise comparisons.

One-way analysis of variance

Data	% of cells
Factor codes	OPN
Sample size	40

Levene's test for equality of error variances

Levene statistic	4.2
DF 1	7
DF 2	32
Significance level	P = 0.002

ANOVA

Source of variation	Sum of Squares	DF	Mean Square
Between groups (influence factor)	4782.0438	7	683.1491
Within groups (other fluctuations)	24.3994	32	0.7625
Total	4806.4432	39	

F-ratio	895.955
Significance level	P < 0.001

Tukey-Kramer test for all pairwise comparisons

Factor	n	Mean	SD	Different (P<0.05) from factor nr
(1) 1	5	64.2605	1.634	(2)(3)(4)(5)(6)(7)(8)
(2) 2	5	76.4996	0.4425	(1)(3)(4)(5)(6)(7)(8)
(3) 3	5	90.2479	1.0122	(1)(2)(5)(6)(7)(8)
(4) 4	5	90.6053	0.9043	(1)(2)(5)(6)(7)(8)
(5) 5	5	92.4425	0.703	(1)(2)(3)(4)(6)(7)(8)
(6) 6	5	95.5333	0.7672	(1)(2)(3)(4)(5)(7)
(7) 7	5	98.442	0.5125	(1)(2)(3)(4)(5)(6)(8)
(8) 8	5	95.9513	0.2146	(1)(2)(3)(4)(5)(7)

Residuals

D'Agostino-Pearson test for Normal distribution	accept Normality (P=0.1739)

Table S5: Data of morphometric analysis of expression of DSPP in PDLSCs cultured under 3D conditions for 14 days. Anova: One-way analysis of variance with Tukey-Kramer test for all pairwise comparisons.

One-way analysis of variance

Data	% of cells
Factor codes	DSPP
Sample size	40

Levene's test for equality of error variances

Levene statistic	9.249
DF 1	7
DF 2	32
Significance level	P < 0.001

ANOVA

Source of variation	Sum of Squares	DF	Mean Square
Between groups (influence factor)	39661.1883	7	5665.884
Within groups (other fluctuations)	35.5414	32	1.1107
Total	39696.7297	39	

F-ratio	5101.326
Significance level	P < 0.001

Tukey-Kramer test for all pairwise comparisons

Factor	n	Mean	SD	Different (P<0.05) from factor nr
(1) 1	5	0.0000	0.0000	(5)(6)(7)(8)
(2) 2	5	0.0000	0.0000	(5)(6)(7)(8)
(3) 3	5	0.0000	0.0000	(5)(6)(7)(8)
(4) 4	5	0.0000	0.0000	(5)(6)(7)(8)
(5) 5	5	32.8352	0.7166	(1)(2)(3)(4)(6)(7)(8)
(6) 6	5	57.9447	2.4188	(1)(2)(3)(4)(5)(7)(8)
(7) 7	5	80.2638	1.3515	(1)(2)(3)(4)(5)(6)(8)
(8) 8	5	61.9832	0.8333	(1)(2)(3)(4)(5)(6)(7)

Residuals

D'Agostino-Pearson test for Normal distribution	reject Normality (P=0.0024)

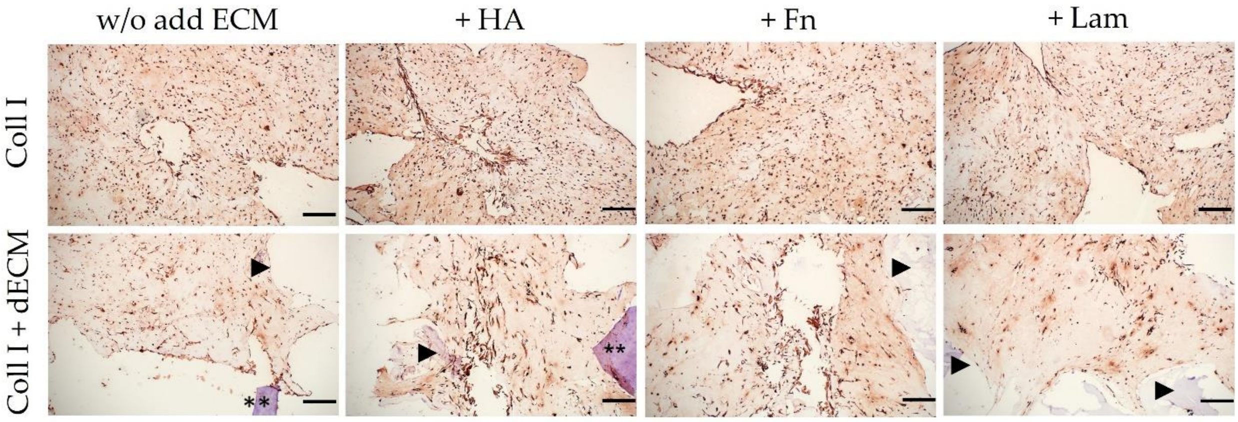

Figure S1: Immunohistochemical staining of PDLSCs for Vim. PDLSCs cultured without scaffolds and with decellularized scaffolds in collagen I hydrogel for 14 days. The positive cells have brown color. The nuclei were counterstained with hematoxylin. Asterisks indicate dTM; arrowheads indicate dPDL. Scale bars, 200 μm.
